# Supplementary material for: Inter-rater reliability of a novel objective endpoint for benign central airway stenosis interventions: Segmentation-based volume rendering of computed tomography scans
Source: PLoS One. 2023 Oct 25;18(10):e0290393. doi: 10.1371/journal.pone.0290393 (PMC10599541; doi:10.1371/journal.pone.0290393)
Supplement: Attachment — Submitted filename: Response to reviewers.docx [file pone.0290393.s001.docx]

Response to reviewers:

Reviewer #1: Many thanks for asking me to review this manuscript.

This is a great study. My only reservations are that the study would have benefited from a larger cohort of patients, with a wider audience (perhaps international) to quantify the variables.

I suspect this study is a pilot study of some sorts - leading to a wider and bigger study.

**Response: We sincerely appreciate the reviewer's careful evaluation of our manuscript. We acknowledge the valid concern raised regarding the small size of our study population. It is important to note that benign airway stenosis is an uncommon disorder, with even major academic centers encountering fewer than 50 patients per year with this diagnosis. In light of this rarity, our data represents a comprehensive summary of patients spanning a 10-year period, thereby highlighting the scarcity of available cases and CT imaging. Furthermore, we would like to emphasize that this study serves as a pilot investigation with two primary objectives. Firstly, it serves as the primary outcome measure it serves as the primary outcome measure for a pilot multicenter randomized controlled trial aimed generate preliminary data on a particular treatment for subglottic stenosis and validate this endpoint. Secondly, it is part of a larger ongoing effort at our institution to prospectively collect data on future patients, with the intention of collaborating with national and international centers in the future.**

Reviewer #2: This paper assesses the inter-rater reliability of a new method of BCAO quantification, comparing it with two conventional visual methods of assessment.

Table 1: indicate which variables are continuous. Also, some percentages appear to be wrongly calculated (for axial slice thickness).

**Response: We thank the reviewer for bringing this to our attention, we have corrected the percentages and have made it more clear which variables are continuous (age).**

Methods: the method requires identifying the nadir stenosis point. Was each rater required to locate this point themselves, or was it specified for them?

**Response: To provide for consistency of measurements between readers, the reader AR marked the nadir point of stenosis on each image to be used as a reference. We have added this explanation in our methods.**

Methods: the method involves measuring +/- 1.5cm above and below the nadir stenosis point. However, some of the volumes appear to have slice thickness >3 mm. How were they handled?

**Response: We apologize for any confusion caused by our previous description. To clarify, the measurement of +/- 1.5cm above and below the nadir stenosis point does not directly pertain to CT slice thickness but rather refers to the spatial extent within the airway that was assessed. CT slice thickness is a distinct technical parameter that determines the thickness of the individual image slices acquired during the CT scan, as mentioned previously. It does not directly impact the measurement technique employed in our study.** **This measurement was performed regardless of the specific CT slice thickness utilized during the scanning process. The purpose was to assess the anatomical characteristics and dimensions of the stenosis within a defined region, irrespective of the thickness of individual slices in the CT dataset.**

Methods: the new method measures absolute volume, but the conventional methods are based on relative cross-sectional area (stenosis severity). I do believe a segmentation method would be more accurate, but a specific formula quantifying stenosis severity from the segmentations should be developed and tested.

**Response: We appreciate the reviewer's valuable comments. We acknowledge the limitations associated with traditional methods, which often pose challenges in terms of accuracy, reproducibility, and ease of use. In light of these considerations, we opted for the segmentation method employed in our study. This approach capitalizes on the utilization of readily available CT images within the clinical workflow, offering the advantage of being easily performed by readers with varying levels of expertise in interpreting airway cross-sectional images. A formula-based approach could possibly be evaluated in future studies.**

Methods and results: the new segmentation method would likely take longer to perform than the conventional visual methods. The time taken for assessment should be measured to understand the trade-off.

**Response: Although the segmentation method may require slightly more time for implementation, its ease of use and straightforward measurement steps have resulted in relatively high agreement among users. Considering this positive outcome, it would be intriguing to explore the aspect of time in future studies. However, we would like to reemphasize the limitations associated with conventional visual methods. These drawbacks encompass poor relative accuracy, limited reproducibility, difficulties in comprehending the stenotic structure in three-dimensional space, challenges in effectively communicating measurements, and limitations in tracking temporal changes over time. By highlighting these issues, we underscore the need for alternative approaches such as the segmentation method employed in our study to overcome these shortcomings and enhance the reliability and comprehensiveness of airway stenosis assessments.**

General: OsiriX is called "open source" in multiple locations in the text. OsiriX may have started off as open source, but its current incarnation is commercial software.

**Response: We appreciate the reviewer's valid observation. However, we would like to clarify that all the measurement components and aspects of our study were conducted using the open-source functionality of OsiriX. No readers involved in our study incurred any expenses for commercial software usage. We ensured that our measurements were performed solely with the freely available tools and features of OsiriX, thereby promoting accessibility and eliminating any potential financial barriers for the readers involved in our research.**
