# Supplementary material for: Inter-rater reliability of a novel objective endpoint for benign central airway stenosis interventions: Segmentation-based volume rendering of computed tomography scans
Source: PLoS One. 2023 Oct 25;18(10):e0290393. doi: 10.1371/journal.pone.0290393 (PMC10599541; doi:10.1371/journal.pone.0290393)
Supplement: Attachment — Submitted filename: Response to reviewers.docx [file pone.0290393.s002.docx]

Response to reviewers:

**Reviewer #2:**

1. To provide for consistency of measurements between readers, the reader AR marked the nadir point of stenosis on each image to be used as a reference. We have added this explanation in our methods.

I understand why this was done, but unfortunately this limits the validity of the interrater measurement, particularly when the paper claims that “readers with minimal training and expertise in airway cross-sectional imaging were able to have strong agreement”. At the least, this should be mentioned in the study limitations.

Also, was a similar reference location specified for the subjective method? Please state it similarly in the text.

**Response: We express our gratitude to the reviewer for their valuable comments. The remark regarding the measurement of the nadair point as a reference is well-taken, and we acknowledge that it may somewhat restrict the validity of the interrater measurements. Nevertheless, this approach was adopted to minimize ambiguity when identifying structural abnormalities, with a primary focus on accurate measurements. However, it is worth noting that this approach was not employed for the subjective method. We will duly acknowledge this limitation in our study.**

2. A formula-based approach could possibly be evaluated in future studies.

The point I'm making is that both methods are measuring different outcomes. The subjective method explicitly assesses stenotic severity (normal vs. stenotic), whereas the objective method only measures total volume, with no assessment of stenosis. E.g., if a particular volume threshold is used to diagnose stenosis, there's no indication how the interrater variability would affect such a diagnosis. At the least, this should be mentioned in the study limitations.

**Response: You are correct in pointing out that both methods in our study are measuring different outcomes. We acknowledge that this issue was not explicitly addressed in the study, and the topic of future work. Your suggestion to include this point in the study limitations is well-founded, and we agree that it is essential to highlight this potential limitation to provide a comprehensive understanding of our findings.**

3. Although the segmentation method may require slightly more time for implementation, its ease of use and straightforward measurement steps have resulted in relatively high agreement among users.

I agree that the higher accuracy would likely be valuable. However, the objective measurements do take more time and the trade-off should be made clear. Anyway, the lack of time measurements should be mentioned in the study limitations.

**Response: You raise an important point regarding the trade-off between higher accuracy and the additional time required for objective measurements. We completely agree that this trade-off should be transparently presented in our study to provide a comprehensive understanding of the implications of using objective measurements.**

4. We would like to clarify that all the measurement components and aspects of our study were conducted using the open-source functionality of OsiriX.

“Open source” carries a specific meaning, that the source code is made available. As far as I’m aware, the last open source version of OsiriX was v5.8 (I think the current version is v12). You should probably use the term “free of charge” instead.

**Response: Valid points, we will clarify and include the term that the version used was “free of charge”.**

**Reviewer #3:**

1. You only reported the imaging evaluation. It would be of great value to compare these results with the endoscopy findings. Do you have these data?

**Response: Thank you for raising a valuable point regarding the comparison of imaging evaluation with endoscopy findings. We understand the significance of such a comparison, as it can provide comprehensive insights into the clinical correlations and validate the accuracy of our imaging assessments. We do not have endoscopic images for most cases in our dataset. This is a common scenario in our study setting, as patients often undergo imaging without undergoing endoscopic intervention (surveillance). As a result, we were unable to directly compare the imaging results with endoscopy findings. This presents an opportunity for future research and correlation of tracheal volume to severity of stenosis on endoscopy. We will note this in our limitations.**

2. In the last paragraph of the section "Statistical analysis", references 18 and 19 should be above the line, as the remaining references of the manuscript. The hyperlink of the website mentioned should also be removed.

**Response: Thank you, we will address this.**
